# Testing the Missing Mechanism of Demographic and Health Variables in the Health and Retirement Study

**DOI:** 10.1093/geroni/igaa057.1644

**Published:** 2020-12-16

**Authors:** Peiyi Lu, Mack Shelley

**Affiliations:** Iowa State University, Ames, Iowa, United States

## Abstract

Studies using data from longitudinal health survey of older adults usually assumed the data were missing completely at random (MCAR) or missing at random (MAR). Thus subsequent analyses used multiple imputation or likelihood-based method to handle missing data. However, little existing research actually examines whether the data met the MCAR/MAR assumptions before performing data analyses. This study first summarized the commonly used statistical methods to test missing mechanism and discussed their application conditions. Then using two-wave longitudinal data from the Health and Retirement Study (HRS; wave 2014-2015 and wave 2016-2017; N=18,747), this study applied different approaches to test the missing mechanism of several demographic and health variables. These approaches included Little’s test, logistic regression method, nonparametric tests, false discovery rate, and others. Results indicated the data did not meet the MCAR assumption even though they had a very low rate of missing values. Demographic variables provided good auxiliary information for health variables. Health measures (e.g., self-reported health, activity of daily life, depressive symptoms) met the MAR assumptions. Older respondents could drop out and die in the longitudinal survey, but attrition did not significantly affect the MAR assumption. Our findings supported the MAR assumptions for the demographic and health variables in HRS, and therefore provided statistical justification to HRS researchers about using imputation or likelihood-based methods to deal with missing data. However, researchers are strongly encouraged to test the missing mechanism of the specific variables/data they choose when using a new dataset.

